# A petroclival glioma mimicking trigeminal schwannoma: A case report

**DOI:** 10.1097/MD.0000000000027792

**Published:** 2021-11-19

**Authors:** Hui-Min Xie, Seidu A. Richard, Zhigang Lan

**Affiliations:** a Department of Radiology, West China Hospital, Sichuan University, 37 Guo Xue Xiang Road, Chengdu, Sichuan, PR China; b Department of Neurosurgery, West China Hospital, Sichuan University, 37 Guo Xue Xiang Road, Chengdu, Sichuan, PR China; c Department of Medicine, Princefield University, P. O. Box MA 128, Ho-Volta Region, Ghana, West Africa.

**Keywords:** extra-axial, glioma, petroclivus, radiotherapy, surgery, temozolamide

## Abstract

**Rationale:**

Glioma in the petroclival region is very rare. Also, very few cases of primary gliomas have been reported to have radiographic as well as intraoperative features of extra-axial lesions resulting in diagnostic dilemma in the literature. We present a rare case of petroclival glioma mimicking trigeminal schwannoma in a young female.

**Patient concerns:**

We present a 21-years old female with a 3-month history of pain in the right eye with no visual impairment. Cranial nerves examination revealed mild deficits in the trigeminal nerve, facial nerve, auditory nerve, oculomotor as well as the trochlear nerve.

**Diagnoses:**

Magnetic resonance imaging detected an extra-axial mass with mixed signal intensities in the right petroclivus area. Immunohistochemical established glioma with world health organization (WHO) grade II.

**Interventions:**

The lesion was resected via 2 successive operations in 6 months interval. The patient was further treated with radiotherapy and post-radiotherapy temozolamide.

**Outcomes:**

Two years follow-up revealed no recurrence of the lesions and she is well. Nevertheless, he is still being followed diligently to uncover any recurrence.

**Lessons:**

The extra-axial nature as well as petroclival location of the glioma makes our case very unique and very rare. The imaging characteristics were very extraordinary for a glioma which resulted in diagnostic dilemma. Thus, the definitive diagnosis was based on the histopathological evaluation of the excised tumor.

## 1 Introduction

Glioma in the petroclival region is very rare and so far, only one case has been reported in literature.^[1]^ Gliomas are the most frequent malignant brain tumor in adults.^[2]^ They are typically detected in the cerebral hemispheres.^[2]^ Very few cases of primary gliomas have been reported to have radiographic as well as intraoperative features of extra-axial lesions resulting in diagnostic dilemma in the literature.^[2]^ The diagnosis of glioma is still very challenging notwithstanding the innovations in imaging modalities.^[1,2]^ Thus, diagnosis is often based on the histopathologic confirmation of the excised tumor. We present a rare case of petroclival glioma mimicking trigeminal schwannoma in a young female.

## 2 Case report

A 21-years old female presented with a 3-month history of pain in the right eye with no visual impairment. She denied association of trauma of any kind. She also denied headaches, nausea and vomiting. She had open cholecystectomy when she was 8 years old. She had no history of hypertension or diabetics. General physical examination was unremarkable. Cranial nerves examination revealed mild deficits in the trigeminal nerve, facial nerve, auditory nerve, oculomotor as well as the trochlear nerve. Ophthalmic examination of both eyes was unremarkable. Routine laboratory investigations were grossly at normal rangers. Routine Chest X-ray and electrocardiogram did not show any abnormalities.

**Figure 1:**
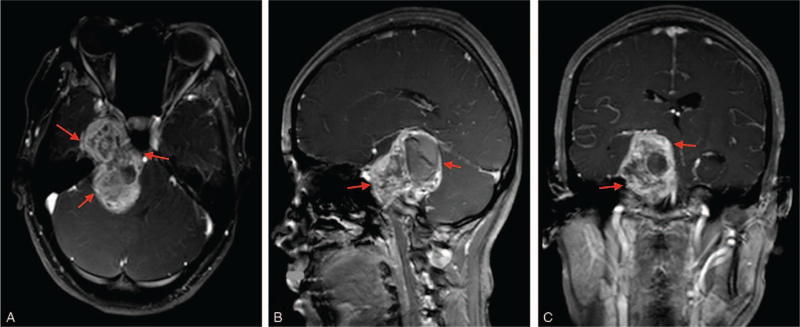
(A & B): Are preoperative MRI showing an extra-axial mass with mixed signal intensities in the right petroclivus. **A** = axial view, **B** = sagittal view, **C** = coronal view*. Red arrow = tumor*.

Magnetic resonance imaging (MRI) detected an extra-axial mass with mixed signal intensities in the right middle and posterior cranial fossa (right petroclivus) measuring 5.6 × 2.8 × 4.7 cm (Fig. 1 A–C). The mass was isointense to hypointense on T1 weighted imaging, hyperintense on T2 weighted imaging as well as hyperintense on FLAIR sequence. The lesion was unevenly enhanced on enhancement scan. The brain stem as well as the third and fourth ventricles were compressed with resorption of adjacent petrous bone. The bilateral paraventricular white matter showed patchy hyperintense on FLAIR. The mass showed multilineage features of trigeminal neuroma with bilateral white matter demyelination.

Based on the MRI findings above, we opted to resect the lesion with massive decompression and preservation of cranial nerves like the trigeminal nerve, facial nerve, auditory nerve, oculomotor as well as the trochlear nerve and repair of sinus defects. The lesion was resected via 2 successive operations in 6 months interval. Electromyographic and auditory brainstem responses were utilized to monitor the cranial nerves above in both operations. We first resected the posterior part of the lesion via the right retrosigmoid approach.

**Figure 2:**
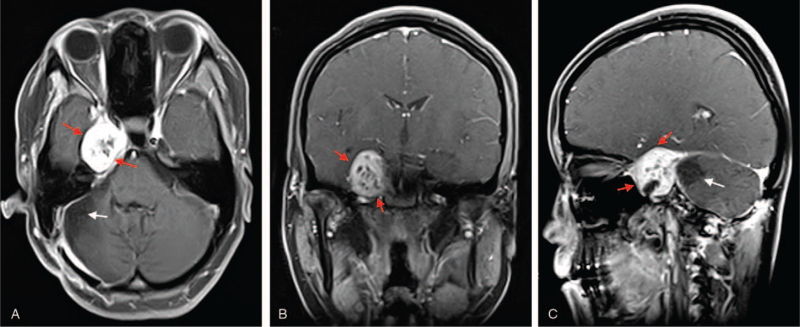
(A–C): Are first postoperative MRI showing tumor in the petrous part but no tumor in the posterior part. **A** = axial view, **B** = coronal view, **C** = sagittal view. *Red arrow = tumor, White = No tumor*.

After general anesthesia, the patient was put on the park bench position with her head fixed in Mayfield 3 keys’ head support system and a right retrosigmoid incision made. Intraoperatively, the tumor was located in the petroclival region, extending from the right tentorium, the upper, middle and lower clivus. The tumor was adhering and compressing the facial nerve, auditory nerve, oculomotor as well as the trochlear nerve. The tumor was carefully separated from these cranial nerves and peripheral vessels. The tumor was yellow in color with clear borders. Also, it was hard in consistency with rich blood supply. The lower clival part of the tumor was completed resected. Postoperative MRI done showed no tumor in the posterior part (Fig. 2A–C). Nevertheless, petrous part of the lesion was visible measuring 2.9 × 2.2 × 1.2 cm with similar feature as describe in the preoperative MRI above (Fig. 2A–C).

**Figure 3:**
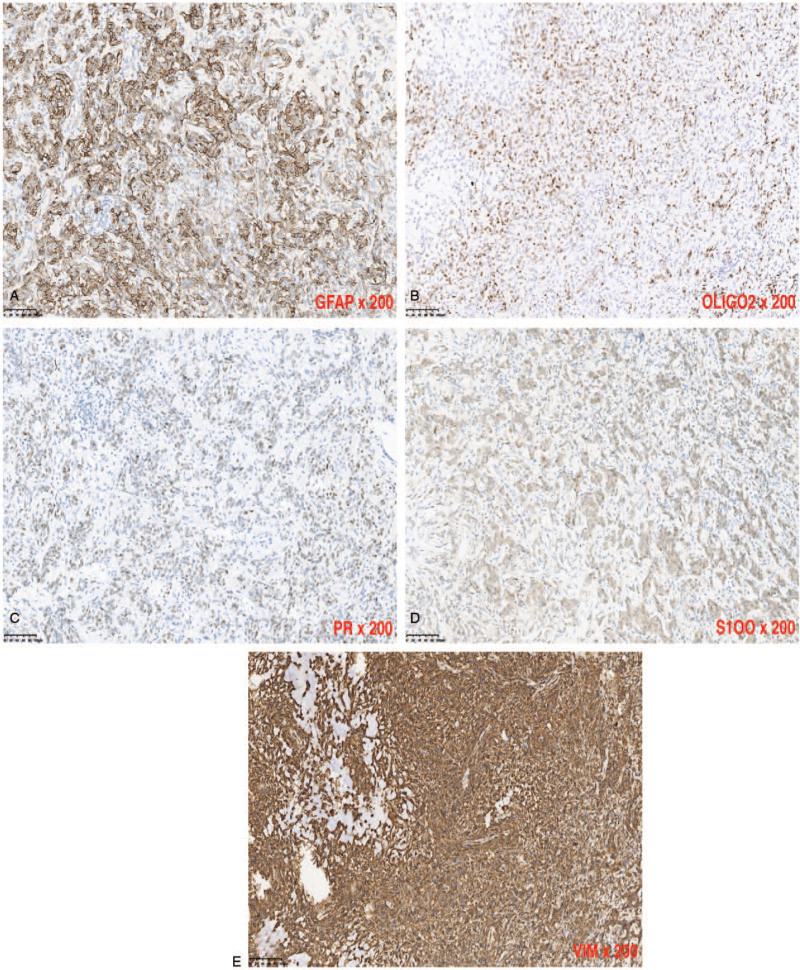
(A–E): Are immunohistochemical staining of the resected lesion revealed positivity for tumor markers like 3A = glial fibrillary acidic protein (GFAP), 3B = oligodendrocyte transcription factor (OLIGO2), 3C = progesterone receptor (PR), 3D = S100, and 3E = Vimentin (VIM).

Immunohistochemical staining of the resected lesion revealed positivity for tumor markers like glial fibrillary acidic protein (GFAP), oligodendrocyte transcription factor, progesterone receptor (PR), S100, vimentin (VIM) as well as Ki-67 labeling index of about 3% (Fig. 3A–E) and negativity for markers like Somatostatin receptor 2, thyroid transcription factor-1, CD34, CD57, H3K27 M, human melanoma black-45, signal transducer and activator of transcription 6 as well as epithelial membrane antigen. These finding were consistent with the diagnosis of glioma: world health organization (WHO) grade II. Postoperative course was uneventful after the operation. She was discharged home 2 weeks later after scheduling the second operation. Postoperative examination before discharge revealed no neurological deficits.

**Figure 4:**
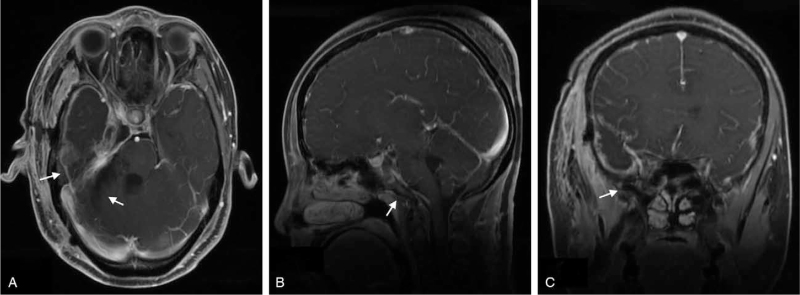
(A–C): Are postoperative MRIs showing no tumor in the petroclival region. **A** = axial view, **B** = sagittal view, **C** = coronal view. *White = No tumor*.

The second operation was carried out 6 months after the first operation via the right anterior transpetrosal approach. Intraoperatively, the tumor was located in the petrosal region. The tumor was adhering and compressing the trigeminal nerve. The tumor was carefully deserted from the trigeminal nerve, the facial and vestibulocochlear nerve and peripheral vessels. The tumor was yellow in color with clear borders. Also, it was hard in consistency with rich blood supply. After securing hemostasis, the dura was closed water tight and bone flap replaced. The muscles and skin were closed in layers. Postoperative MRI done showed no tumor in the petroclival region (Fig. 4A–C).

Immunohistochemical staining of the second samples revealed positivity for GFAP, oligodendrocyte transcription factor, PR, S100, VIM as well as Ki-67 labeling index of about 3% which was same as the first immunohistochemical report above and negativity for H3K27 M, pancytokeratin, thyroid transcription factor-1, integrase interactor-1. Also, inhibitor of differentiation protein 1-1/2 gene mutations were not detected. Furthermore, no mutations were detected in telomerase reverse transcriptase gene and no methylation was detected in the promoter regions. These second finding were also consistent with the diagnosis of glioma: WHO grade II.

Postoperative examination before discharge revealed no neurological deficits and no cranial nerve deficits. The patient was discharged home 2 weeks after admission in the hospital. The patient was further treated with radiotherapy and postradiotherapy temozolamide (TMZ) at our hospital's oncology department. Two years follow-up revealed no recurrence of the lesions and she is well. Nevertheless, he is still being followed diligently to uncover any recurrence.

## 3 Discussion

Glioma in the petroclaval region is very rare and so far, only Karthigeyan et al have reported a case.^[1]^ Glioma with prime extra-axial features has rarely been described in the literature.^[3]^ Most gliomas are detected in the elderly compared to our patient who was relatively younger.^[1,2]^ Our case is very unique and very rare because, the lesion was extra-axial and located at petroclivus. Furthermore, the imaging characteristics were very extraordinary for a glioma which resulted in diagnostic dilemma. The clinical presentation in our case is of sudden onset of pain in the right as well as mild neurological deficits in cranial nerves like trigeminal nerve, facial nerve, auditory nerve, oculomotor as well as the trochlear nerve.

Karthigeyan et al who saw a similar case observed that, the lesion was extra-axial type in the left petroclival on computed tomography and was heterogeneously hypodense with chunky foci of calcification.^[1]^ On MRI, they further observed an extra-axial lesion which was isointense to hypointense on T1-weighted imaging and hyperintense on T2-weighted imaging with moderate postcontrast enhancement.^[1]^ Our MIR findings were analogous to Karthigeyan et al MRI findings. We resected the lesion via 2 successive operations in 6 months interval because of the diagnostic dilemma of the lesion on radiology. We first resected the posterior part of the lesion via the right retrosigmoid approach and pathological evaluation of the resected samples gave us a clue of the nature of the lesion. The second operation was carried out 6 months after the first operation via the right anterior transpetrosal approach.

Several surgical approaches have been used to resect lesions in the petroclival region.^[4,5]^ The retrosigmoid approach also referred to as retromastoid or lateral suboccipital approach is the most frequently used technique in resecting lesions at the cerebellopontine angle as well as the petroclival regions.^[4–6]^ This approach involves bone removal angled on the asterion borders of the transverse as well as sigmoid sinuses and their junction.^[4–6]^ The dura is often resected in an arch manner and reflected outward, concealing the exposed sinuses.^[4–6]^ We utilized this approach in our first operation because the tumor was entirely extra-axial and more revealing at posterior fossa. Also, this exposure allowed for maximum preservation of the cranial nerves.

Anterior transpetrosal approach or the “Shiobara-Kawase approach” is also used to access tumors at the petroclival region.^[4,7,8]^ This approach provides an ideal extradural view of the clival region while exposing the trigeminal nerve, the facial and vestibulocochlear nerve complexes.^[4,7]^ This approach is often a completely extradural approach with very reduced retraction on the temporal lobe.^[4,7,8]^ We utilized this approach in our second operation because the tumor was entirely extra-axial and this time round, more anterior after resecting the posterior part of the tumor. Furthermore, this exposure allowed for maximum preservation of the cranial nerves.

The transpetrosal approach with variants such as presigmoid, posterior petrosectomy, retrolabyrinthine, transcrusal as well as transcochlear approaches have been described.^[4,9,10]^ The transpetrosal approach is often utilized to resect tumors that invades the middle as well as upper third of petroclival area which mostly involve the petrous bone.^[4,9,10]^ The craniectomy commence with a complete mastoidectomy so that the middle fossa as well as posterior fossa dura are well exposed.^[4,9,10]^ This exposure permits visualization of the transverse-sigmoid junction as well as the exposure of the superior petrosal sinus.^[4,9,10]^

Most recently, endoscopic endonasal approaches to the petroclival region are substitutes for all ventral open approaches like facial degloving, transfacial, Le Fort osteotomies, as well as transbasal approaches.^[4,11,12]^ This approach allows for the same or enhanced exposure with minimum morbidity for the patient.^[4,11,12]^ Usually, this approach to the petroclival area necessitates a transpterygoid approach.^[4,11,12]^ Treat of WHO grade II comprises of utmost safe resection of the tumor followed by radiotherapy with postradiotherapy TMZ chemotherapy.^[13]^

Stupp et al indicated that daily simultaneous treatment with temozolomide at 75 mg/m^2^ as an adjunct to radiotherapy of 30 × 2 Gy equivalent to 60 Gy at tumor bed followed by up to 6 cycles of temozolomide at 150 to 200 mg/m^2^ on 5 of out 28 days prolonged median overall survical compared to radiotherapy alone.^[14]^ Based in these studies, we further treated the patient with radiotherapy and postradiotherapy TMZ at our hospital's oncology department as per the retreatment regime above.

Immunohistochemical analysis is very useful in differentiating the various types gliomas as well as their subtypes.^[15]^ The most crucial marker for detection of glial differentiation is GFAP.^[15,16]^ Virtually all gliomas secret S-100 protein, making it a sensitive marker for these malignancies.^[15]^ Nevertheless, S-100 secretion is a relatively nonspecific glioma marker because a variety tumors secrete it.^[15]^ OLIG2 does not permit for obvious differentiation between oligodendrogliomas and diffuse or pilocytic astrocytomas. It is however very useful in the differential diagnosis of a diffuse glioma and a neuronal, ependymal, or nonglial tumor type because OLIG2 staining is characteristically restricted or deficient in the latter.^[15,17]^

There is usually an upsurge in the Ki-67 labeling index in diffuse gliomas with malignancy grade of about <5% in low-grade diffuse gliomas, 5% to 10% in anaplastic gliomas, as well as >10% in glioblastomas.^[15,18]^ Initial, the WHO grade I encompass pilocytic astrocytoma's, grade II were low-grade astrocytoma's (astrocytoma grade I and II), grade III were anaplastic astrocytoma's (astrocytoma grade III) while grade IV were glioblastoma multiform (astrocytoma grade IV).^[19]^

In the 2016 classification, the diffuse gliomas comprised of the WHO grade II and grade III astrocytic tumors, the grade II and III oligodendrogliomas, the grade IV glioblastomas, as well as the related diffuse gliomas of childhood.^[20]^ The WHO grade II diffuse astrocytomas as well as WHO grade III anaplastic astrocytomas are now respectively separated into IDH-mutant, IDH-wildtype as well as NOS types.^[20]^ It is specified that, if immunohistochemistry for mutant R132H IDH-1 protein and sequencing for IDH-1 codon 132 as well as IDH-2 codon 172 gene mutations are both negative, or if sequencing for IDH-1 codon 132 as well as IDH-2 codon 172 gene mutations alone is negative, then the lesion can be identified as IDH wildtype.^[20]^ In our patient, inhibitor of differentiation protein 1-1/2 gene mutations were not detected. Also, no mutations were detected in telomerase reverse transcriptase gene and no methylation was detected in in the promoter regions.

## 4 Conclusion

The extra-axial nature as well as petroclival location of the glioma makes our case very unique and very rare. The imaging characteristics were very extraordinary for a glioma which resulted in diagnostic dilemma. Thus, the definitive diagnosis was based on the histopathological evaluation of the excised tumor. Treat of WHO grade II comprises of utmost safe resection of the tumor followed by radiotherapy with post-radiotherapy TMZ chemotherapy.

## Author contributions

**Conceptualization:** Hui-Min Xie, Seidu A Richard, Zhigang Lan.

**Data curation:** Hui-Min Xie, Seidu A Richard, Zhigang Lan.

**Formal analysis:** Hui-Min Xie, Seidu A Richard, Zhigang Lan.

**Funding acquisition:** Zhigang Lan.

**Investigation:** Hui-Min Xie, Seidu A Richard, Zhigang Lan.

**Methodology:** Hui-Min Xie, Seidu A Richard, Zhigang Lan.

**Resources:** Zhigang Lan.

**Supervision:** Zhigang Lan.

**Writing – original draft:** Seidu A Richard.

**Writing – review & editing:** Hui-Min Xie, Seidu A Richard, Zhigang Lan.
